# Prediction of marginal adaptation failure in restorations of non-carious cervical lesions, based on machine learning

**DOI:** 10.1590/1807-3107bor-2025.vol39.106

**Published:** 2025-10-10

**Authors:** Thalita de Paris Matos BRONHOLO, Pedro Felipe de Jesus FREITAS, Aline Xavier FERRAZ, Kaliane Rodrigues da CRUZ, Michael Willian FAVORETO, Flares BARATTO-FILHO, Alessandra REIS, Alessandro Dourado LOGUERCIO, Cristiano Miranda de ARAUJO

**Affiliations:** (a) Universidade Tuiuti do Paraná, School of Dentistry, Curitiba, PR, Brazil.; (b) Universidade Tuiti do Paraná, Postgraduate Program in Human Communication Health, Curitiba, PR, Brazil.; (c) Universidade da Região de Joinville – Univille, School of Dentistry, Joinville, SC, Brazil.; (d) Universidade Estadual de Ponta Grossa – UEPG, School of Dentistry, Department of Restorative Dentistry, Ponta Grossa, PR, Brazil.

**Keywords:** Machine Learning, Dental Marginal Adaptation, Composite Resins, Dental Restoration Failure

## Abstract

Marginal adaptation failure in noncarious cervical restorations (NCCRS) significantly compromises restoration longevity and adversely impacts patient outcomes. early identification of high-risk restorations is therefore of clinical importance. This study aimed to develop and evaluate a supervised machine learning (ML) model capable of predicting the risk of marginal adaptation failure in NCCRS within 18 months following treatment. A total of 262 restorations were analyzed, incorporating multiple clinical variables, including adhesive system used, cavity geometry, degree of dentin sclerosis, incisogingival height, tooth characteristics, and patient age. Seven supervised ml algorithms were trained and assessed: decision tree, support vector machine (SVM), gradient boosting, k-nearest neighbors (KNN), logistic regression, multilayer perceptron, and random forest. model performance was evaluated using fivefold cross-validation and standard metrics, including the area under the receiver operating characteristic curve (AUC), accuracy, recall, precision, and F1 score. Key predictive features identified were incisogingival height, patient age, and type of adhesive system. the auc values ranged from 0.72 (95% confidence interval [95%CI]: 0.57–0.88) to 0.52 (95%CI: 0.51–0.83), and recall values ranged from 0.77 (95%CI: 0.66–0.89) to 0.53 (95%CI: 0.40–0.66). Among the tested algorithms, SVM, gradient boosting, and KNN demonstrated superior predictive performance. these findings suggest that ml models can serve as effective tools for predicting restoration failure and may assist clinicians in optimizing post-treatment monitoring and follow-up strategies for patients with NCCRS.

## Introduction

Noncarious cervical lesions (NCCLs) are frequently encountered in clinical dental practice and are characterized by the progressive loss of hard tissue at the cervical region of teeth, without the involvement of dental caries or traumatic injury.^1^ Composite resin restorations are widely used for the management of NCCLs due to their favorable esthetic properties and mechanical performance. Recent advances in restorative materials and application protocols have contributed to enhanced clinical outcomes by promoting better integration with the surrounding dentition.^2^

A key determinant of restoration longevity is the quality of marginal adaptation. Optimal marginal sealing not only prevents microleakage and bacterial infiltration but also improves fracture resistance, supports periodontal health, and enhances esthetic outcomes.^3-6^ However, clinical evidence indicates that marginal adaptation failures often manifest within 18 months following restoration placement,^3,7-9^ emphasizing the importance of identifying predictive factors to mitigate early failure.

Effective marginal adaptation minimizes the potential for biofilm accumulation, reduces the likelihood of secondary caries, and contributes to the long-term success of restorations.^4^ The quality of the adhesive bond plays a pivotal role and is influenced by variables such as the degree of dentin sclerosis, which may compromise adhesion^11,12^and flexural stress arising from cervical margin positioning.^11^ Even minimal interfacial gaps can increase susceptibility to secondary caries,^10^ making early prediction of marginal adaptation failure critical to clinical success.^13^

In recent years, the application of artificial intelligence (AI) in healthcare has grown substantially, enabling more individualized and accurate prognostic evaluations. Machine learning (ML), a subfield of AI, utilizes mathematical algorithms trained on structured datasets to make predictive inferences.^14^ To date, no published studies have used ML techniques to predict the risk of marginal adaptation failure in NCCL restorations. Developing such a model could significantly improve risk stratification and clinical monitoring for patients undergoing restorative treatment.

Therefore, the present study aimed to develop and validate a supervised ML-based predictive model to assess the risk of marginal adaptation failure in NCCL restorations within the first 18 months post-treatment.

## Methods

### Study design and setting

This study utilized data derived from previously conducted randomized, controlled clinical trials employing a double-blind, split-mouth, and equivalence design. The trials were conducted between August 2014 and November 2016 at dental clinics affiliated with two universities in southern Brazil. All trials were approved by the respective Scientific Review Boards and the Ethics Committees for the Protection of Human Participants (approval numbers: 2.888.374; 2.974.728; 3.893.891; 3.056.864; 4.825.578; 5.017.516; 5.375.918 and 5.972.183). Additionally, the trials were registered on recognized clinical trial platforms (registration numbers: RBR-86pszd; RBR-38s2x9; RBR-7t7d4d; RBR-4hcvsg; RBR-274vf96; RBR-9k8ymkm; RBR-5bj464v and RBR-6jpgyjr), ensuring adherence to international ethical guidelines.^15, 16^

### Participants

Participants were included based on the following criteria: a) age ≥18 yr; b) general systemic health; c) acceptable oral hygiene status as defined by the Simplified Oral Hygiene Index^17^; d) presence of at least 20 teeth in occlusion; and e) at least two adjacent NCCLs on vital teeth with similar size, shape, and depth. Eligible lesions were non-retentive, involved both enamel and dentin, exhibited a depth > 1 mm, and were located on teeth without mobility. Furthermore, at least 50% of the lesion margins had to be within the enamel.

### Exclusion criteria included inadequate oral hygiene, current use of orthodontic appliances, advanced or chronic periodontitis, severe bruxism, known allergies to materials used in the study, and current pregnancy or lactation.

**Data collection**

Randomization procedures, blinding protocols, sample size estimation, and restorative techniques were performed according to previously published methodologies.^15,16^All clinical data used to construct the predictive model were collected by experienced and calibrated examiners.

Evaluator training included the analysis of 10 representative images per evaluation criterion. These images depicted cervical restorations but were not drawn from the actual study sample. Data collection commenced only after achieving a minimum intra- and inter-examiner agreement of ≥85%, as measured by the κ coefficient.^18^

### Study variables

Clinical data were collected at two time points: baseline and 18 months post-restoration. Each examiner independently assessed restorations using a standardized paper form. Discrepancies between examiners were resolved through consensus before finalizing the evaluation.

The following variables were recorded:

- **Marginal adaptation**: Evaluated based on criteria from the World Dental Federation (FDI).^6, 19^ Restorations were rated on a scale ranging from clinically very good to clinically poor, with replacement indicated for the latter. A marginal gap ≥ 50 µm or a minor marginal fracture removable by polishing was considered an early sign of misfit and required closer monitoring. Scores of 2, 3, 4, and 5 were classified as potential adaptation failures.
- **Adhesive system**: Classified as either a one-step self-etch system (1-step SE) or a conventional two-step etch-and-rinse system (2-step ER).
- **Cavity geometry**: Categorized according to the internal angle of the lesion: <45°, 45°–90°, 90° to <135°, and >135°.^20^
- **Dentin sclerosis**: Assessed using a four-point sclerosis scale: 1 (none), 2 (mild), 3 (moderate), and 4 (severe).^21,22^
- **Incisogingival height**: Measured as the vertical distance from the occluso-incisal edge to the cervical margin of the lesion. Lesions were grouped into four categories: < 1.5 mm, 1.5–2.5 mm, 2.5–4.0 mm, and > 4.0 mm.
- **Tooth characteristics**: Classified according to dental arch (maxillary or mandibular) and tooth type (incisor, canine, premolar, or molar).
- **Patient age**: Self-reported age at the time of the restorative procedure.
- All clinical evaluations were performed after dental prophylaxis using pumice paste and water applied with a rubber cup.

### Data analysis and model construction

Predictor variable selection for each model was performed using recursive feature elimination with cross-validation (RFECV). This method systematically removes the least informative features while reassessing model performance at each iteration, thereby identifying the most relevant variables for optimal ML accuracy.

To address class imbalance, the Synthetic Minority Over-sampling Technique (SMOTE) was applied exclusively to the training dataset, increasing the representation of the minority class (i.e., restoration failures). SMOTE was implemented using Python’s imbalanced-learn library to avoid information leakage from the test set, thereby improving model robustness for rare outcome prediction.

Tree, Random Forest, Gradient Boosting Classifier, Logistic Regression, KNN, Multilayer Perceptron (MLP) Classifier, and Support Vector Machine (SVM).

Hyperparameter optimization for each algorithm was conducted using grid search with fivefold cross-validation. A broad yet computationally feasible hyperparameter space was defined to ensure optimal tuning of each model. All steps—including preprocessing, feature selection, model training, tuning, and evaluation—were executed using Python with the *scikit-learn* library. The complete implementation is publicly available at DOI: 10.5281/zenodo.12772908.

### Training, cross-validation, and test splitting

**Figure 1:**
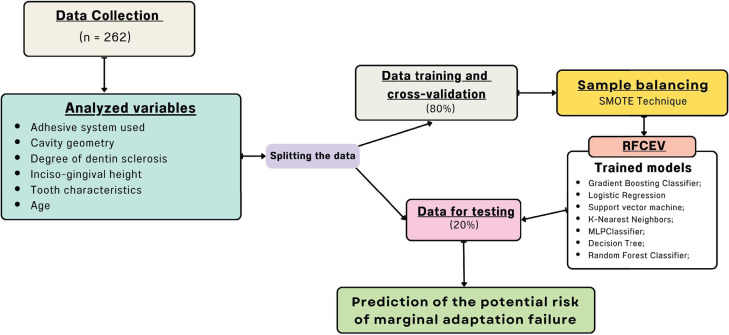
Flowchart diagram illustrating the process of data analysis employing machine learning models.

The dataset was randomly divided into training/cross-validation (80%) and independent test (20%) sets using the train_test_split function from the sklearn.*model_selection* module. Model generalizability was assessed using fivefold cross-validation, wherein the dataset was partitioned into five equal subsets; in each fold, four subsets were used for training and one for validation. This process was repeated five times, and the average validation performance was computed. The complete modeling workflow is illustrated in Figure 1.

### Model evaluation metrics

Model performance was assessed using the receiver operating characteristic (ROC) curve, which plots the true positive rate against the false positive rate across various classification thresholds. This evaluation was based on predicted probabilities for the positive class, derived from the test set, and their corresponding true labels.

The area under the ROC curve (AUC) was calculated using the roc_auc_score function from the *scikit-learn* library. To provide a comprehensive assessment of classification performance, additional evaluation metrics were computed for each model:

- Accuracy: Indicates overall classification efficiency, calculated as the ratio of correct predictions to the total number of samples:

$$\text{ Accuracy }=\frac{\text{ True positives }+\text{ True negatives }}{\text{ Total elements }}$$

- Precision: Measures the proportion of true positive predictions among all positive predictions:

$$\text{ Precision }=\frac{\text{ True positives }}{\text{ True positives }+\text{ False positives }}$$

- Recall (Sensitivity): Reflects the model’s ability to identify actual positive cases:

$$\text{ Recall }=\frac{\text{ True positives }}{\text{ True positives }+\text{ False negatives }}$$

- F1 Score: Represents the harmonic mean of precision and recall, providing a balanced measure of model performance:

$$\text{ F1 Score }=2*\frac{{\text{ precision }}^{*}\text{ recall }}{\text{ precision }+\text{ recall }}$$

To identify influential variables, model-specific feature importance techniques were employed. For tree-based models (Decision Tree, Random Forest, and Gradient Boosting), the feature_importances_ attribute from *scikit-learn* was used to quantify each variable’s contribution to impurity reduction. For logistic regression, the absolute values of the model coefficients (coef_) served as indicators of variable importance.

Because KNN, MLP, and SVM (with an RBF kernel) do not inherently provide feature importance metrics, these models were excluded from this analysis. Variables were considered most relevant if they consistently ranked as highly important in at least three of the four interpretable models. Selection criteria included both the frequency of appearance and the magnitude of assigned importance.

Ninety-five percent confidence intervals (95% CIs) were calculated for all performance metrics using two methods. For the independent test set, the bootstrap method with 1,000 iterations was applied, and 95% CIs were defined by the 2.5th and 97.5th percentiles of the resulting distribution. For the fivefold cross-validation results, 95% CIs were derived from metric values across the fivefolds to estimate performance variability.

Algorithm performance was compared using bootstrap resampling of the AUC values. Differences between models were deemed statistically significant if the 95% CI of the AUC difference did not include 0.

## Results

A total of 262 restorations from 47 patients were analyzed, with follow-up data collected 18 months after restorative treatment. The sample consisted of 55.3% female and 44.7% male participants, with a mean age of 50.0 ± 9.81 yr. The characteristics of the NCCLs are detailed in Table 1.

**Table 1:** Characteristics of the combination of primary studies of NCCLs.10,11

Characteristics of NCCLs	n (%)
Marginal adaptation	
Possibility of failure	65 (24.8)
Non-failure	197 (75.2)
Shape (degree of angle)	
< 45	0 (0)
45–90	87 (33.2)
90–135	67 (25.6)
> 135	108 (41.2)
Cervico-incisal height (mm)	
< 1.5	44 (16.8)
1.5–2.5	104 (39.7)
2.5–4.0	94 (35.9)
> 4.0	20 (7.6)
Degree of sclerotic dentin	
1	31 (11.8)
2	112 (42.7)
3	96 (36.6)
4	23 (8.8)
Tooth distribution	
Incisor	29 (11.1)
Canines	30 (11.5)
Premolar	170 (64.9)
Molar	33 (12.6)
Arch distribution	
Maxillary	135 (51.5)
Mandibular	127 (48.5)

**Figure 2:**
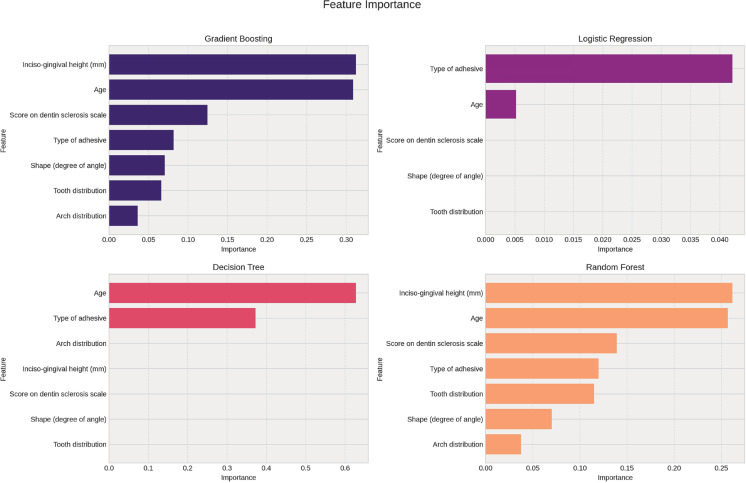
Results of feature importance analysis from four machine learning models.

Based on the ML analyses, the most important predictors of marginal adaptation failure were the incisogingival lesion height, patient age, and the adhesive system used (Figure 2).

The models demonstrated varying levels of performance in predicting marginal adaptation failure. On the test set, the area under the ROC curve (AUC)—a measure of discriminative ability—ranged from 0.72 (95%CI: 0.57–0.88) to 0.52 (95%CI: 0.51–0.83). These results indicate that while some models were able to distinguish between failing and stable restorations with up to 72% accuracy, others performed only marginally better than random guessing.

Recall values, indicating the proportion of true failures correctly identified, ranged from 0.77 (95% CI: 0.66–0.89) to 0.53 (95%CI: 0.40–0.66) on the test set. This suggests that the most effective models successfully detected between 53% and 77% of actual failures, with higher-recall models offering better sensitivity in identifying high-risk cases.

**Figure 3:**
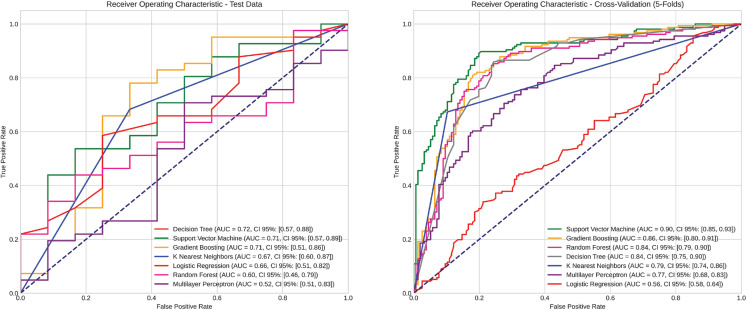
Evaluation of Classification Models using ROC Curves.

During the cross-validation procedure, which evaluated model generalizability, AUC values were generally higher, ranging from 0.90 (95%CI: 0.85–0.93) to 0.56 (95%CI: 0.58–0.64), indicating more stable performance across training partitions. Cross-validation recall values ranged from 0.81 (95%CI: 0.71–0.87) to 0.54 (95%CI: 0.45–0.64; Figure 3).

**Figure 4:**
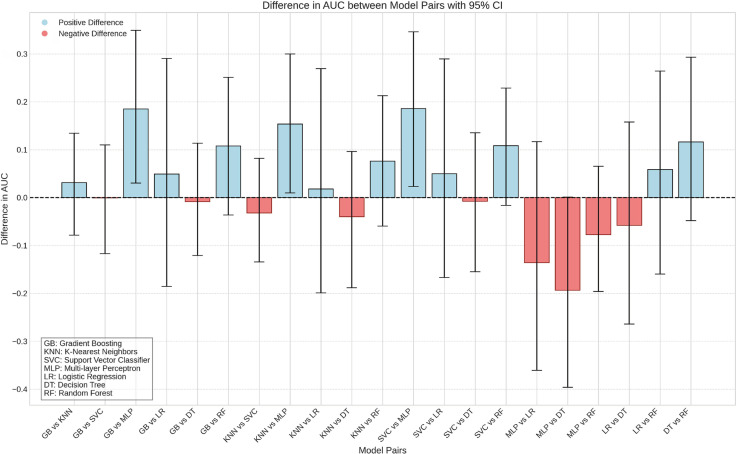
Comparison of the mean difference in AUC between the different algorithms tested, with the respective 95% confidence interval.

Comparison of AUC values revealed statistically significant performance differences among models. Specifically, the SVM, Gradient Boosting, and KNN models outperformed the MLP model, which exhibited the lowest predictive accuracy on the test set (Figure 4).

Comprehensive performance metrics and hyperparameter configurations for both the test and cross-validation sets are provided in Table 2.

**Table 2:** Overview of metrics acquired during the cross-validation and testing phases of the models, alongside their optimal hyperparameters.

Model	Optimal hyperparameters	Cross-validation results [95%CI]	Test data results [95%CI]
Logistic regression	C: 0.05	Accuracy = 0.54 [0.45–0.64]	Accuracy = 0.52 [0.39–0.66]
	max_iter: 100	Precision = 0.49 [0.23–0.64]	Precision = 0.74 [0.58–0.87]
	penalty: l1	Recall = 0.54 [0.45–0.64]	Recall = 0.53 [0.40–0.66]
	l1_ratio: 0.2	F1-Score = 0.49 [0.29–0.63]	F1-Score = 0.56 [0.42–0.69]
	solver: saga		
Gradient boosting classifier	n_estimators: 100	Accuracy = 0.80 [0.73–0.88]	Accuracy = 0.77 [0.66–0.88]
	learning_rate: 0.2	Precision = 0.80 [0.74–0.88]	Precision = 0.78 [0.65–0.90]
	min_samples_split: 2	Recall = 0.80 [0.73–0.88]	Recall = 0.77 [0.66–0.89]
	max_depth: 5	F1-Score = 0.80 [0.73–0.88]	F1-Score = 0.72 [0.65–0.89]
	min_samples_leaf: 4		
K-Nearest neighbors	n_neighbors: 1	Accuracy = 0.78 [0.72–0.86]	Accuracy = 0.68 [0.55–0.79]
	weights: uniform	Precision = 0.81 [0.76–0.88]	Precision = 0.77 [0.64–0.89]
	leaf_size: 100	Recall = 0.78 [0.72–0.86]	Recall = 0.68 [0.55–0.79]
	p: 5	F1-Score = 0.78 [0.71–0.86]	F1-Score = 0.73 [0.59–0.82]
Support vector machine	kernel: rbf	Accuracy = 0.81 [0.70–0.87]	Accuracy = 0.70 [0.57–0.81]
	C: 50	Precision = 0.82 [0.74–0.87]	Precision = 0.74 [0.60–0.87]
	gamma: auto	Recall = 0.81 [0.71–0.87]	Recall = 0.70 [0.57–0.81]
		F1-Score = 0.81 [0.70–0.87]	F1-Score = 0.71 [0.59–0.83]
MLP classifier	activation: than	Accuracy = 0.71 [0.59–0.79]	Accuracy = 0.64 [0.51–0.77]
	alpha: 1.0	Precision = 0.71 [0.59–0.79]	Precision = 0.70 [0.54–0.84]
	hidden_layer_sizes: 1000	Recall = 0.71 [0.59–0.79]	Recall = 0.64 [0.51–0.77]
	learning_rate_init: 0.01	F1-Score = 0.71 [0.59–0.79]	F1-Score = 0.66 [0.52–0.79]
	max_iter: 1000		
	solver: lbfgs		
Decision tree	criterion: gini	Accuracy = 0.80 [0.71–0.87]	Accuracy = 0.70 [0.58–0.81]
	max_depth: none	Precision = 0.80 [0.72–0.87]	Precision = 0.78 [0.66–0.89]
	splitter: best	Recall = 0.80 [0.72–0.87]	Recall = 0.70 [0.58–0.81]
		F1-Score = 0.80 [0.72–0.87]	F1-Score = 0.72 [0.61 - 0.83]
Random forest classifier	max_depth: 7	Accuracy = 0.80 [0.73–0.85]	Accuracy = 0.62 [0.49–0.74]
	n_estimators: 50	Precision = 0.80 [0.74–0.86]	Precision = 0.63 [0.46–0.79]
	min_samples_split: 2	Recall = 0.80 [0.73–0.85]	Recall = 0.62 [0.49–0.74]
	min_samples_leaf: 1	F1-Score = 0.80 [0.73–0.85]	F1-Score = 0.62 [0.48–0.74]
	criterion: gini		
	max_features: auto		

## Discussion

Marginal adaptation failure remains a major concern in composite resin restorations, as it can result in the formation of gaps at the tooth–restoration interface. Such gaps promote microleakage of oral fluids, postoperative sensitivity, and an increased risk of secondary caries.^23^ The present study aimed to develop a supervised ML model capable of predicting marginal adaptation failure, potentially even in its early stages, within 18 months, using characteristics of NCCLs and the adhesive system employed. The goal was to enable early detection and improve monitoring for patients at elevated risk of this complication. Regular follow-up and appropriate maintenance are crucial for the long-term success of restorative treatments.^4,24,25^ To the best of our knowledge, no prior studies have reported ML models specifically designed to predict marginal adaptation failure in NCCLs.

Machine learning, a branch of AI, enables outcome prediction from datasets by identifying patterns and optimizing decision-making processes.^14^ However, one common challenge in biomedical applications—particularly in binary classification tasks such as failure versus non-failure—is data imbalance, as failure events are typically underrepresented.^26,27^ This imbalance can hinder model accuracy and generalizability. In this study, most restorations remained stable at 18 months, reflecting such an imbalance. Nonetheless, model performance varied, with AUC values reaching 0.90 in cross-validation and 0.72 on the test set. The SVM model exhibited relatively balanced performance across metrics, likely due to its ability to effectively handle moderately imbalanced datasets. By focusing on support vectors and reducing the influence of distant negative instances, the SVM model maintained strong predictive capacity.^28^ Moreover, the use of oversampling methods such as SMOTE, which increases the representation of the minority class, likely contributed to improved model performance.^26^

Among the evaluated variables, incisogingival height, patient age, and adhesive type emerged as the most relevant predictors across the tested models. Even shallow NCCLs—as small as 0.5 mm—can result in stress concentration in the cervical region, with stress magnitude increasing proportionally with lesion size.^29-31^ Both external and internal occlusal loading generate heightened stress at the apex of the lesion, compared with its coronal and gingival walls, suggesting that NCCLs may contribute to restoration failure by intensifying stress at the margins.^29-31^ Restoration with resin composites has been shown to dissipate these stresses, significantly improving fracture resistance, approaching that of sound teeth, regardless of loading direction.^32^ This effect is likely due to the elimination of the sharp lesion apex and the use of composite materials with an elastic modulus similar to dentin, allowing for a more uniform distribution of occlusal forces.^32^

Regarding adhesive strategies, evidence suggests that bonding effectiveness is not solely determined by the adhesion protocol. Previous studies have reported comparable risks of marginal discoloration, marginal degradation, retention loss, and fractures when comparing etch-and-rinse with selective enamel-etching approaches.^33,34^ Patient age also plays a critical role in adhesion and marginal integrity, as enamel and dentin undergo morphological and compositional changes over time.^4,35,36^ With aging, root surface exposure increases, dentin becomes more susceptible to wear, and both the prevalence and severity of NCCLs rise.^29^ Notably, universal adhesives have shown acceptable clinical outcomes in NCCL restoration.^37^

This study has several limitations. The 18-month follow-up period is relatively short, and the dataset size was modest, which may limit the generalizability and restrict predictions to the short term. Additionally, the low incidence of marginal adaptation failure may have affected model performance, even with the application of oversampling. Nonetheless, the use of high-quality data derived from randomized clinical trials enhances reliability and minimizes bias.^36^ All data were collected in accordance with standardized FDI criteria, which facilitate consistent classification of restorations based on material, extent, and intraoral location.^5,6,25^

To improve model robustness and minimize overfitting or underfitting, fivefold cross-validation, hyperparameter tuning using Grid Search, and feature selection via RFECV were applied. SMOTE was restricted to the training set to preserve the integrity of the test data. Additionally, bootstrapping with 1,000 iterations was used to estimate 95%CIs for key performance metrics, strengthening the reliability of the findings.

From a clinical standpoint, the predictive model developed in this study may aid in the early identification of patients at risk for marginal adaptation failure in NCCL restorations. Such predictive insights could inform individualized follow-up schedules and maintenance protocols, including shorter recall intervals, personalized oral hygiene recommendations, and optimized material selection. This approach may reduce complications associated with marginal failure, such as microleakage, postoperative sensitivity, and recurrent caries, potentially enhancing restoration longevity. Furthermore, integrating predictive tools into dental practice management systems could support evidence-based, data-driven decision-making in restorative care.

Future studies should aim to extend follow-up beyond 18 months to capture a higher number of failure events, thereby addressing limitations posed by class imbalance and enhancing model training. Expanding the dataset to include a broader range of clinical variables may further improve predictive accuracy. Finally, external validation across different populations and clinical settings is essential to ensure the model’s applicability and generalizability in diverse dental care environments.

## Conclusion

The supervised ML models demonstrated promising predictive performance for identifying marginal adaptation failure in composite resin restorations within 18 months. Among the tested algorithms, the SVM, Gradient Boosting, and KNN models yielded the most favorable results. This data-driven approach offers a valuable tool to support post-treatment monitoring of patients with NCCLs, enabling more personalized, preventive, and proactive clinical care.

## Data Availability

The contents underlying the research text are contained in the manuscript.
